# Enhancing flavonoid production by systematically tuning the central metabolic pathways based on a CRISPR interference system in *Escherichia coli*

**DOI:** 10.1038/srep13477

**Published:** 2015-09-01

**Authors:** Junjun Wu, Guocheng Du, Jian Chen, Jingwen Zhou

**Affiliations:** 1 Key Laboratory of Industrial Biotechnology, Ministry of Education, School of Biotechnology, Jiangnan University, 1800 Lihu Road, Wuxi, Jiangsu 214122, China; 2 Synergetic Innovation Center of Food Safety and Nutrition, 1800 Lihu Road, Wuxi, Jiangsu 214122, China

## Abstract

The limited supply of intracellular malonyl-CoA in *Escherichia coli* impedes the biological synthesis of polyketides, flavonoids and biofuels. Here, a clustered regularly interspaced short palindromic repeats (CRISPR) interference system was constructed for fine-tuning central metabolic pathways to efficiently channel carbon flux toward malonyl-CoA. Using synthetic sgRNA to silence candidate genes, genes that could increase the intracellular malonyl-CoA level by over 223% were used as target genes. The efficiencies of repression of these genes were tuned to achieve appropriate levels so that the intracellular malonyl-CoA level was enhanced without significantly altering final biomass accumulation (the final OD_600_ decreased by less than 10%). Based on the results, multiple gene repressing was successful in approaching the limit of the amount of malonyl-CoA needed to produce the plant-specific secondary metabolite (2S)-naringenin. By coupling the genetic modifications to cell growth, the combined effects of these genetic perturbations increased the final (2S)-naringenin titer to 421.6 mg/L, which was 7.4-fold higher than the control strain. The strategy described here could be used to characterize genes that are essential for cell growth and to develop *E. coli* as a well-organized cell factory for producing other important products that require malonyl-CoA as a precursor.

Malonyl-coenzyme A (malonyl-CoA) is an important precursor metabolite involved in the biosynthesis of many important chemicals, such as flavanones^1^, polyketides^2^ and fatty acids^3^,^4^. However, in most microorganisms commonly used for the heterologous production of these chemicals, such as *Escherichia coli* and *Saccharomyces cerevisiae*, the endogenous central metabolism predominates and strongly competes for carbon sources and energy during malonyl-CoA synthesis, leaving only small amounts available for the production of recombinant products^1^,^5^,^6^,^7^. Nevertheless, previous studies have made some exciting gains in improving the intracellular synthesis of malonyl-CoA through overexpression or deletion of specific genes^1^,^5^,^8^.

For example, overexpression of four acetyl-CoA carboxylase (ACC) subunits and biotin ligase from *Photorhabdus luminescens*^9^ or the malonate assimilation pathway from *Rhizobium trifolii*^10^,^11^ have significantly improved the availability of malonyl-CoA. However, overexpression of these genes would impose metabolic burdens on the cells and often requires supplementation of biotin or malonate in the medium, which is commercially unfavorable. Simultaneous deletion of the genes *sdhA* (succinate dehydrogenase), *adhE* (acetaldehyde dehydrogenase), *brnQ* (branched chain amino acid transporter) and *citE* (citrate lyase)^8^ or *fumC* (fumarate hydratase) and *sucC* (succinyl-CoA synthetase)^1^ also enhanced the malonyl-CoA concentration. While numerous metabolic engineering targets, such as genes essential for growth, are yet to be explored due to the disadvantages of conventional gene-knockout strategies. Furthermore, there has been no effort to evaluate the combinatorial effects of various distinct genetic interventions including those involving genes essential for growth.

The use of RNA-mediated regulatory mechanisms for fine flux control has been well documented^12^. Due to the inefficiency of RNA interference (RNAi) in bacteria^13^,^14^, the clustered regularly interspaced short palindromic repeats interference (CRISPRi) system offers a potential alternative for targeted gene regulation in bacteria, as it uses small base-pairing RNAs to regulate gene expression in a sequence-specific manner^15^. Compared with other sRNA strategies^16^,^17^, the CRISPRi system can easily tune gene expression levels by directing sgRNA to different regions of the non-template DNA strand of target genes^15^. It is believed that this RNA-guided genome regulation can identify unprecedented genetic perturbations and explore the combinatorial effects of multiple genetic manipulations.

Flavonoids are valuable natural products widely used in human health and nutrition due to their biochemical properties, which include antiviral, anti-obesity and anti-cancer activities^18^,^19^. (2S)-Naringenin is a common precursor of most flavonoids^20^ and has become a potential candidate for treating numerous human maladies^18^. Formation of (2S)-naringenin from L-tyrosine occurs through the action of tyrosine ammonia lyase (TAL), 4-coumarate:CoA ligase (4CL), chalcone synthase (CHS) and chalcone isomerase (CHI). Every flavanone compound generated in *E. coli* requires 3 mol of malonyl-CoA, which imposes a significant metabolic burden on the host strain^8^. Previous approaches to redirecting endogenous malonyl-CoA into heterologous pathways have relied heavily on overexpression or deletion of particular pathways^1^,^5^,^8^.

Here, we explored the impact of fine-tuning the central metabolic pathways by using a CRISPRi system^15^ to enhance heterologous pathway productivity, using the production of (2S)-naringenin as a model system. Genes involved in central metabolic pathways were repressed by the CRISPRi system^15^ to identify individual target genes that could increase the intracellular malonyl-CoA level by over 223%. Furthermore, the efficiency of repression of these target genes was tuned to balance malonyl-CoA generation and biomass accumulation (the final OD_600_ decreased by less than 10%). Finally, multiple gene repressing was performed to achieve a high yield of (2S)-naringenin (421.6 mg/L). This strategy enhances the malonyl-CoA concentration without the need to add substrates for malonyl-CoA generation, which potentially provides an economically sustainable process for the efficient production of other plant natural compounds.

## Results

### Construction of the CRISPRi system to perform genetic perturbations

**Figure 1:**
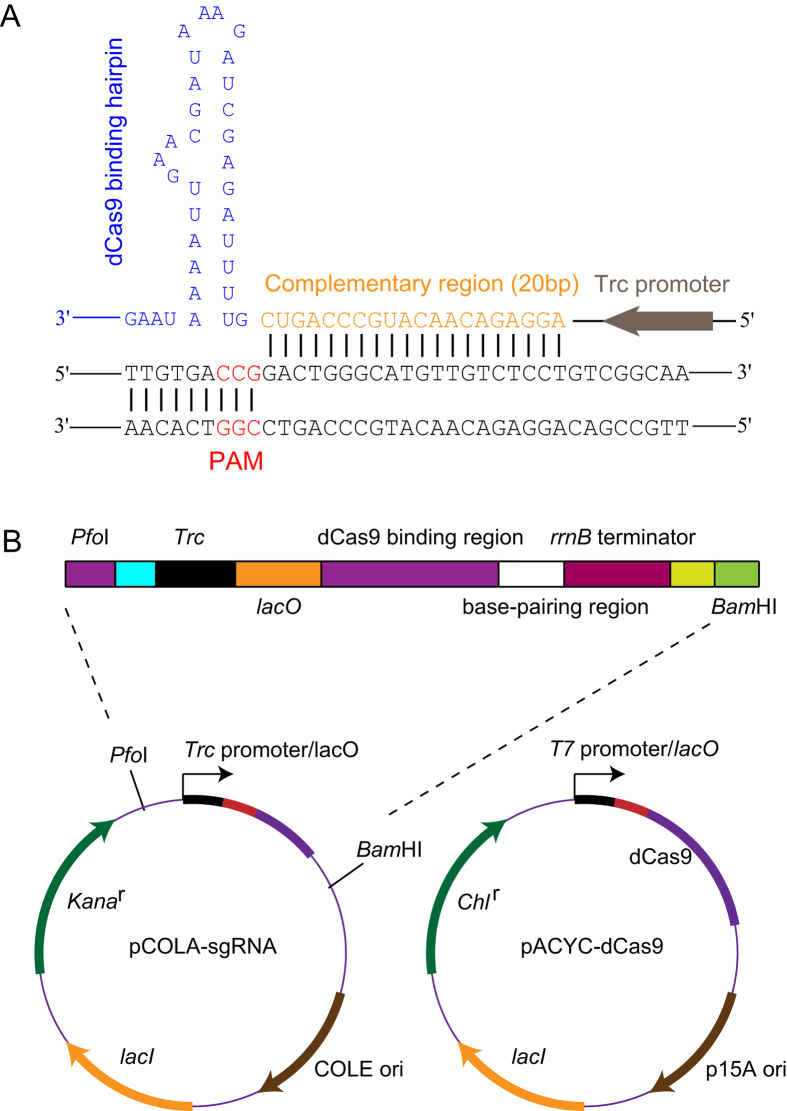
Construction of the CRISPRi system for controlling gene expression. (**A**) Sequence of the designed sgRNA template. sgRNA targets the non-template DNA strand of the gene-coding region. Base-pairing nucleotides (20 bp) are shown in orange. The dCas9-binding hairpin is in blue. The PAM sequence is shown in red. The *Trc* promoter is shown in grey. (**B**) This CRISPRi system consists of an inducible dCas9 protein and a designed sgRNA chimera. The dCas9 mutant gene contains two silencing mutations of the RuvC1 and HNH nuclease domains. The sgRNA chimera contains four functional domains: a *Trc*-inducible promoter, a 20-nucleotide (nt) complementary region for specific DNA binding, a 42-nt dCas9-binding hairpin and a 40-nt transcription terminator derived from *S. pyogenes*^15^.

To implement the CRISPRi platform in *E*. *coli*, a catalytically dead Cas9 mutant (dCas9) derived from *Streptococcus pyogenes cas9* gene^15^, which acts as an RNA-guided DNA-binding complex, was expressed under *T7* promoter. The sgRNA molecule, which consists of four domains (a *Trc* promoter, a 20-nucleotide (nt) complementary region for specific DNA binding, a 42-nt dCas9-binding hairpin and a 40-nt transcription terminator derived from *S. pyogenes*), was coexpressed with dCas9^15^ (Fig. 1).

### Identification of malonyl-CoA related genes by the CRISPRi system

**Figure 2:**
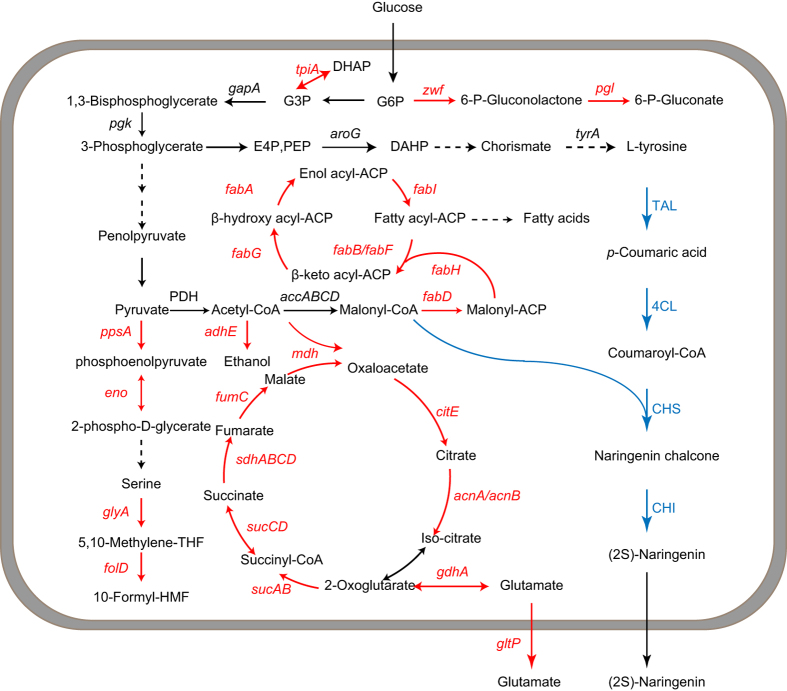
Screening genetic targets to redirect the carbon flux toward malonyl-CoA. Genes selected for efficient channeling of the carbon flux toward malonyl-CoA are shown in red. The metabolic pathway that performs heterologous biosynthesis of (2S)-naringenin from L-tyrosine in *E. coli* is shown in blue. CHI: chalcone isomerase; CHS: chalcone synthase; 4CL: 4-coumarate:CoA ligase; E4P: erythrose-4-phosphate; PEP: phosphoenolpyruvate; TAL: tyrosine ammonia lyase.

Acetyl-CoA serves as the first flux control point for flavonoid biosynthesis, whereas malonyl-CoA serves as a starting point for the synthesis of flavonoids, which are only consumed for synthesizing fatty acids^5^. To channel carbon flows toward acetyl-CoA, the availability of acetyl-CoA precursors such as pyruvate needs to be increased and hence, *zwf* (glucose-6-phosphate 1-dehydrogenase), *pgl* (6-phosphogluconolactonase), *tpiA* (triosephosphate isomerase), *ppsA* (phosphoenolpyruvate synthase), *eno* (phosphopyruvate hydratase), *glyA* (serine hydroxymethyltransferase) and *fold* (bifunctional 5,10-methylene-tetrahydrofolate dehydrogenase/5,10-methylene-tetrahydrofolate cyclohydrolase) were chosen as target genes. Consumption of acetyl-CoA in other central metabolic pathways, such as the TCA cycle and glycolysis, needs to be reduced and hence, *mdh* (malate dehydrogenase), *fumC*, *sdhABCD* (succinate dehydrogenase), *sucCD* (succinyl-CoA synthetase), *sucA* (2-oxoglutarate dehydrogenase), *sucB* (dihydrolipoamide acetyltransferase), *acnAB* (aconitate hydratase), *citE* (citrate lyase), *gdhA* (glutamate dehydrogenase), *gltp* (glutamate/aspartate:proton symporter) and *adhE* (acetaldehyde dehydrogenase) were also chosen (Fig. 2). Fatty acid biosynthesis related genes, such as *fabH* (3-oxoacyl-acyl carrier protein synthase III), *fabB* (3-oxoacyl-acyl carrier protein synthase I), *fabF* (3-oxoacyl-acyl carrier protein synthase II), *fabG* (3-oxoacyl-acyl carrier protein reductase), *fabA* (3-hydroxydecanoyl-acyl carrier protein dehydratase), *fabI* (enoyl-acyl carrier protein reductase) and *fabD* (malonyl-CoA-acyl carrier protein transacylase) were chosen as target genes (Fig. 2) and were also inhibited to prevent the diversion of malonyl-CoA to fatty acid synthesis.

The efficacy of silencing is inversely correlated with the distance of the target gene from the translation start codon^15^. A sequence of designed sgRNA firstly targeted the non-template DNA strand of a specific gene after the first protospacer adjacent motif (PAM) sequence (NGG) at the open reading frame (ORF). To identify potential genes related to the enhanced supply of intracellular malonyl-CoA and acetyl-CoA, thus confirm the efficiency of CRISPRi based technique, the changing pattern of intracellular malonyl-CoA and acetyl-CoA concentration were measured after silencing each target gene.

**Figure 3:**
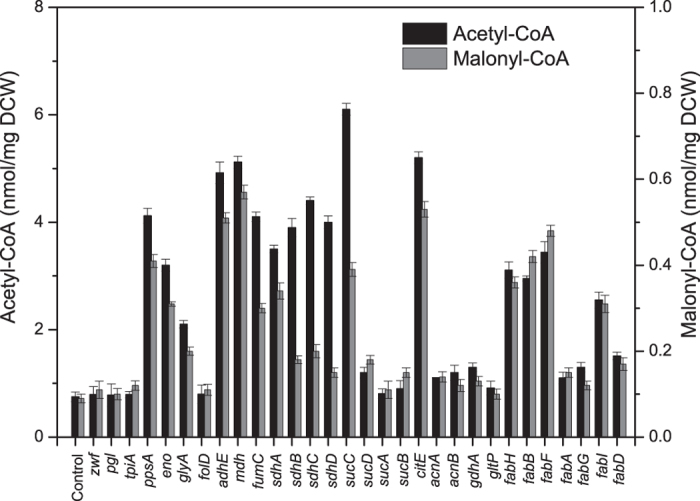
A CRISPRi-based strategy for targeting gene identification. All target genes were silenced with high efficacy. It was found that sgRNAs targeting *ppsA*, *eno*, *glyA*, *adhE*, *mdh*, *fumC*, *sdhABCD*, *sucC*, *cite*, *fabH*, *fabB*, *fabF* and *fabI* showed dramatic increases in acetyl-CoA concentration (increased by over 180%). Meanwhile, sgRNAs targeting *ppsA*, *eno*, *adhE*, *mdh*, *fumC*, *sdhA*, *sucC*, *cite*, *fabH*, *fabB*, *fabF* and *fabI* showed dramatic increases in acetyl-CoA and malonyl-CoA concentrations (increased by over 223%). 1 mL of cell culture was harvested at the mid-log phase of growth to quantify the intracellular concentrations of malonyl-CoA and acetyl-CoA.

The results showed that sgRNAs targeting *ppsA*, *eno*, *glyA*, *adhE*, *mdh*, *fumC*, *sdhABCD*, *sucC* and *citE* produced a dramatic increase in acetyl-CoA concentration (over 180%), while sgRNAs targeting *ppsA*, *eno*, *adhE*, *mdh*, *fumC*, *sdhA*, *sucC* and *citE* produced a dramatic increase in malonyl-CoA concentration (over 223%). It was also found that sgRNAs targeting *fabH*, *fabB*, *fabF* and *fabI* produced a simultaneous increase in acetyl-CoA and malonyl-CoA concentration (over 244%) (Fig. 3). Hence, *ppsA*, *eno*, *adhE*, *mdh*, *fumC*, *sdhA*, *sucC*, *citE*, *fabH*, *fabB*, *fabF* and *fabI* were chosen as target genes.

### Coordination of cell growth and malonyl-CoA accumulation

Acetyl-CoA and malonyl-CoA are important metabolic intermediates involved in central metabolic pathways, such as glycolysis, the TCA cycle^21^ and fatty acid biosynthesis^4^,^22^,^23^. Reducing the acetyl-CoA or malonyl-CoA pool available to these pathways would decrease cell growth. To examine whether fine-tuning target gene expression levels using the CRISPRi system could balance the metabolic flux between cell growth and malonyl-CoA accumulation, three sgRNA variants with different efficiencies for repressing every target gene were generated.

**Figure 4:**
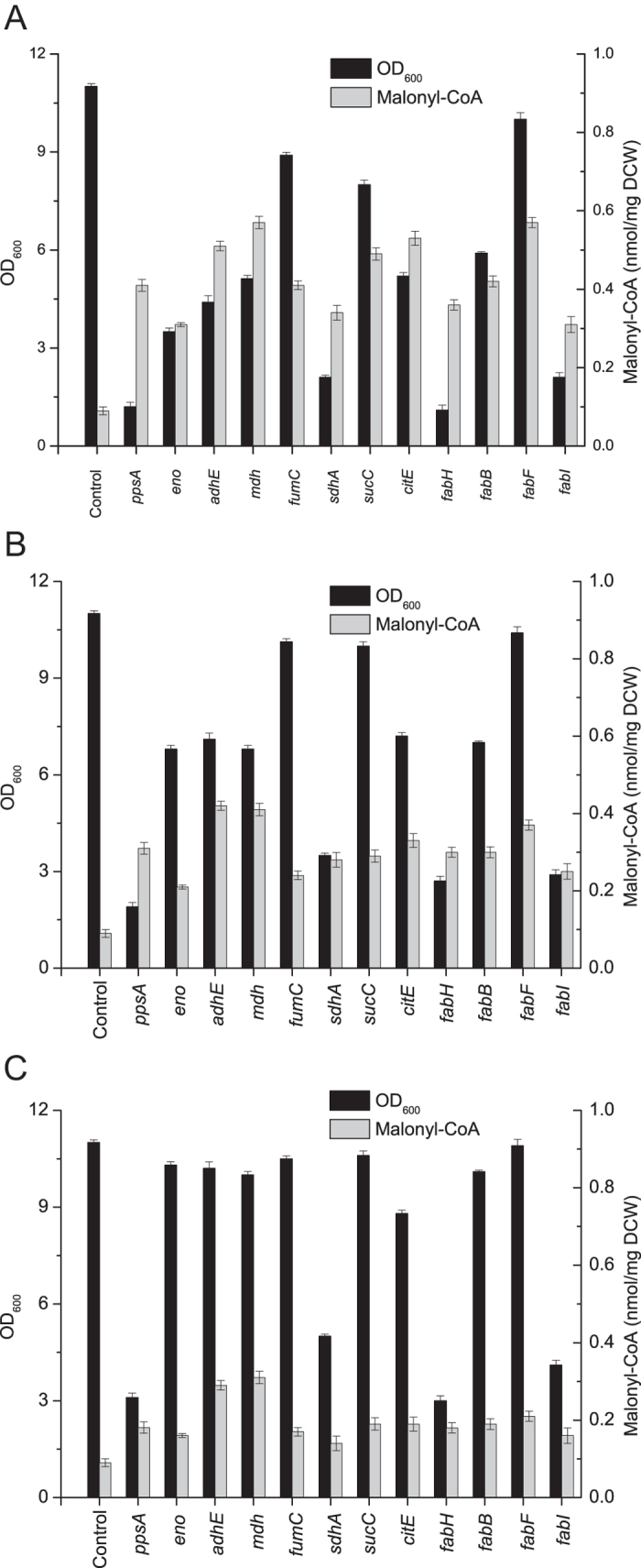
Tuning target gene expression to balance cell proliferation and malonyl-CoA accumulation. To achieve high, medium or low silencing efficacy toward each target gene, sgRNA bound the non-template DNA strand of the target gene at the initial, intermediate or terminal gene coding region. (**A**) Target genes were repressed with high silencing efficacy. (**B**) Target genes were repressed with medium silencing efficacy. (**C**) Target genes were repressed with low silencing efficacy. 1 mL of cell culture was harvested at the mid-log phase of growth to quantify the intracellular concentration of malonyl-CoA. Final OD_600_ values of cultures were measured after a total fermentation time of 48 h.

To achieve a high, medium or low repressing efficacy toward each target gene, sgRNA was designed to bind the non-template DNA strand of the target gene at the initial, middle or terminal region^15^. High repressing efficacy toward *fabF* increased the malonyl-CoA concentration by 433.3% and decreased the final biomass by 9% (Fig. 4). Medium repressing efficacy toward *sucC* and *fumC* increased the malonyl-CoA concentration by 222.4% and 166.8% and decreased the final biomass by 9.1% and 7.9%, respectively. Low repressing efficacy toward *eno*, *adhE*, *mdh* and *fabB* increased the malonyl-CoA concentration by 77.8%, 222.2%, 244.4% and 111.1% and decreased the final biomass by 6.4%, 7.3%, 9.9% and 8.2%, respectively (Fig. 4). SgRNAs targeting other genes resulted in dramatic decreases in the final biomass (by over 54.5%) (Fig. 4). Hence, *eno*, *adhE*, *mdh*, *fabB*, *fabF*, *sucC* and *fumC* were chosen as the optimal target genes.

### Effects of single genetic perturbations on (2S)-naringenin production

**Figure 5:**
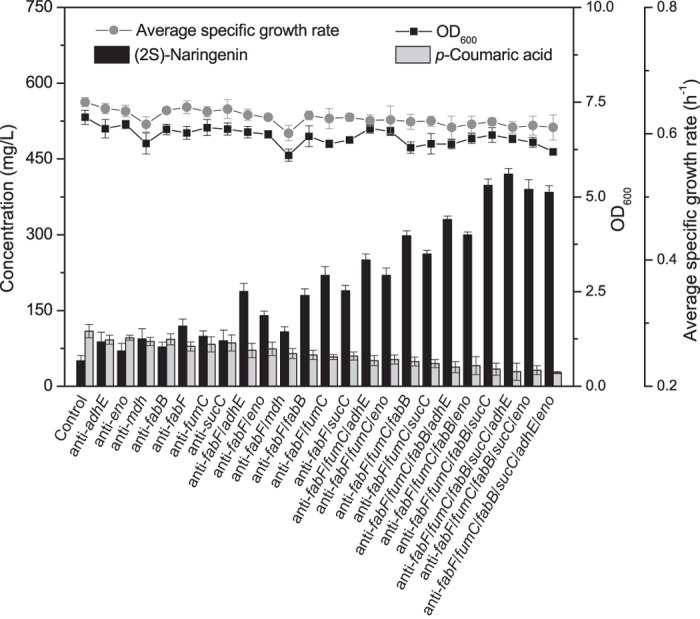
Effects of single or multiple genetic perturbations on (2S)-naringenin production. Control strains contained the (2S)-naringenin heterologous pathway without an RNA-guided dCas9:sgRNA system. The sgRNA-expressing plasmids repressing single or multiple genes were further transformed into the control strain to investigate the effects of these systems on (2S)-naringenin production. Final OD_600_ values, average specific growth rates and concentrations of *p*-coumaric acid and (2S)-naringenin were measured from production strains after a total fermentation time of 48 h.

Based on a previous study^24^, the control strain overproducing (2S)-naringenin from L-tyrosine was constructed. The plasmids pCDF-Trc-TAL-Trc-4CL and pET-CHS-CHI^24^ were transformed into *E. coli* BL21 (DE3), which yielded a production titer of 50.5 mg/L. Appropriate repressing efficacy toward a target gene that could increase the malonyl-CoA concentration without significantly altering the final biomass was ranked as a beneficial genetic perturbation. The impacts of these independent genetic interventions, namely low repressing efficacy toward *eno*, *adhE*, *mdh* and *fabB*, medium repressing efficacy toward *sucC* and *fumC* and high repressing efficacy toward *fabF*, on (2S)-naringenin production were analyzed. Single perturbations of *eno*, *adhE*, *mdh*, *fabB*, *fabF*, *sucC* and *fumC* increased production by up to 38.6%, 74.3%, 86.1%, 54.5%, 135.6%, 78.2% and 96.1%, respectively, and perturbation of *fabF* resulted in the highest (2S)-naringenin production (119.6 mg/L) (Fig. 5).

### Effects of combinatorial genetic perturbations on (2S)-naringenin production

The impacts of different synthetic sgRNA combinations on (2S)-naringenin production were evaluated. Firstly, the effect of two genetic perturbations on (2S)-naringenin production was investigated. Anti-*fabF* sgRNA in combination with anti-*eno*, anti-*adhE*, anti-*fabB*, anti-*sucC* or anti-*fumC* sgRNA but not anti-*mdh* produced a higher (2S)-naringenin titer than single anti-*fabF* sgRNA. The latter result is probably due to an imbalance between cell growth and product formation because it was observed that the strain anti-*fabF*/*mdh* resulted in the lowest cell growth (Fig. 5). Among these sgRNA combinations, anti-*fabF*/*fumC* sgRNA produced the highest (2S)-naringenin titer (220.1 mg/L).

Secondly, the effect of anti-*fabF*/*fumC* sgRNA in combination with anti-*eno*, anti-*adhE*, anti-*fabB* or anti-*sucC* sgRNA was investigated. Each sgRNA combination produced a higher (2S)-naringenin titer than anti-*fabF*/*fumC*, and anti-*fabF*/*fumC*/*fabB* produced the highest (2S)-naringenin titer (298.8 mg/L) (Fig. 5). Similarly, anti-*fabF*/*fumC*/*fabB* sgRNA in combination with anti-*eno*, anti-*adhE* or anti-*sucC* sgRNA produced a higher (2S)-naringenin titer than anti-*fabF*/*fumC*/*fabB* sgRNA, and anti-*fabF*/*fumC*/*fabB*/*sucC* sgRNA produced the highest (2S)-naringenin titer (398.3 mg/L). Anti-*fabF*/*fumC*/*fabB*/*sucC* sgRNA in combination with anti-*eno*, or anti-*adhE* sgRNA produced a higher (2S)-naringenin titer than anti-*fabF*/*fumC*/*fabB*/*sucC* sgRNA, and anti-*fabF*/*fumC*/*fabB*/*sucC*/*adhE* sgRNA produced the highest (2S)-naringenin titer (421.6 mg/L). However, anti-*fabF*/*fumC*/*fabB*/*sucC*/*adhE*/*eno* sgRNA produced a lower (2S)-naringenin titer (384.5 mg/L) than anti-*fabF*/*fumC*/*fabB*/*sucC*/*adhE* sgRNA (Fig. 5).

## Discussion

Identification of potential genetic targets and optimization of their expression are essential for the efficient production of desired metabolites^16^. However, the number of genes that can be manipulated through the traditional gene disruption approach is limited, especially when target genes are critical for growth^25^. There is an urgent requirement for a tool that enables genome-scale identification of suitable gene targets for rational engineering. In this study, the CRISPRi system^15^,^26^ was employed to efficiently regulate the expression of targeted genes involved in central metabolic pathways, such as glycolysis, the TCA cycle or fatty acid synthesis (Fig. 3). The strategy reported here is advantageous compared to conventional gene-knockout strategies for large-scale screening and manipulating genes essential for growth.

There has been increasing interest in the use of RNA-mediated regulatory mechanisms for target gene regulation^16^,^25^. Previously, antisense RNA was employed to simultaneously repress *fabB* and *fabF* to enhance the intracellular malonyl-CoA concentration^11^. However, numerous metabolic engineering targets are yet to be explored and there has been no effort to evaluate the combinatorial effects of various distinct genetic interventions. Here, genes involved in central metabolic pathways were explored to identify target genes. Furthermore, combinatorial effects of various genetic interventions were evaluated to obtain the best combination.

Previous studies have made significant gains in improving the intracellular malonyl-CoA concentration and achieved a high yield of flavonoids through: (1) the ACC route, which requires coordinated overexpression of four acetyl-CoA carboxylase (ACC) subunits from *Photorhabdus luminescens* and biotin ligase from the same species^1^,^9^; (2) the malonate assimilation pathway, which requires the malonate assimilation pathway from *Rhizobium trifolii*^10^,^11^. However, overexpression of these genes would impose a metabolic burden on cells and often requires supplementation of biotin or malonate in the medium, which is commercially unfavorable. In this study, metabolic circuits were rewired by multiple and simultaneous perturbations, through reducing flux to undesirable by-products and enhancing precursor and cofactor flux, the target compound (2S)-naringenin production without adding biotin or malonate was maximizing.

In many cases, simultaneous modification of the expression levels of many genes is necessary to improve the production of target molecules^27^. However, previous studies have found that the impact of multiple genetic interventions on production is not additive. Simple stacking of genetic interventions led to only limited increases in recombinant production^1^,^8^. Here, by coupling genetic modification to cell growth, it was found that, except for repressing *mdh* which resulted in the lowest cell growth, the combined effects of other genetic modifications on production were additive (Fig. 5). This demonstrated that coupling genetic modification to cell growth when scoring the fitness of a genotype is critical to identifying beneficial interventions.

Although malonyl-CoA is the limiting factor for flavonoid production, many studies have demonstrated that balance in the synthetic pathway is vital for productivity^28^,^29^. Accumulation of intermediate metabolites may result in suboptimal product titers, as further increases in these intermediates cannot be accommodated by the capacity of the downstream pathway^29^. Here, it was found that combining all of the positive genetic interventions resulted in a decrease in (2S)-naringenin production compared to other combinations (Fig. 5). Therefore, systematically repressing target genes to approach the limit of malonyl-CoA saturation generates the optimal result.

In conclusion, this CRISPRi system is advantageous over traditional gene-knockout strategies because of its ability to manipulate genes essential for growth. Furthermore, because of its high specificity when regulating a single gene or multiple genes, this RNA-guided system is more productive than antisense RNA strategies. Moreover, the CRISPRi strategy, which couples genetic modification to cell growth to identify beneficial interventions and systematically combines genetic interventions to approach the limit of malonyl-CoA saturation, could be employed for the efficient production of many other malonyl-CoA derived compounds, such as the flavonoids, fatty acids and polyketides.

## Methods

### Strains, plasmids and general techniques

*E. coli* JM109 was used for plasmid propagation. *E. coli* BL21 (DE3) was used to express sgRNA and produce flavonoids. The compatible vector set (pETDuet-1, pCDFDuet-1, pCOLADuet-1, and pACYCDuet-1) was used to express multiple genes in one strain (Novagen, Darmstadt, Germany)^30^. Luria broth (LB) (10 g/L tryptone, 5 g/L yeast extract and 10 g/L NaCl) was used for strain construction. MOPS minimal medium^31^ supplemented with 5 g/L glucose and an additional 4 g/L NH_4_Cl was used for sgRNA expression and flavonoid production. Ampicillin (100 μg/mL), kanamycin (40 μg/mL), chloramphenicol (20 μg/mL), and streptomycin (40 μg/mL) was added when required. Cell growth was monitored by measuring the absorbance at 600 nm (OD_600_) with a UV/Vis spectrophotometer (UVmini-1240, Shimadzu, Kyoto, Japan). The primers and plasmids used in this study are listed in Tables 1, 2 and 3.

### Construction of sgRNA-expressing plasmids to repress a single gene

A catalytically dead Cas9 mutant (dCas9)^15^ was codon-optimized and synthesized by GenScript (Nanjing, China) (see Supplementary Sequences for the dCas9 sequence). pACYC-dCas9 was constructed by digesting dCas9 from pUC57-dCas9 (GenScript, Nanjing, China) into *Nco*I/*Hin*dIII sites of pACYCDuet-1. The sgRNA chimera, which consists of four domains (a *Trc*-inducible promoter, a 20-nucleotide (nt) complementary region for specific DNA binding, a 42-nt dCas9-binding hairpin and a 40-nt transcription terminator derived from *Streptococcus pyogenes*^15^) was synthesized by GenScript (Nanjing, China) and inserted into *pfo*I/*Bam*HI sites of pCOLADuet-1 (see Supplementary Sequences for sgRNA chimera sequence). This resulted in the plasmid pCOLA-fabF(high), which was the template for PCR-based mutagenesis. PCR-based site-directed mutagenesis (TaKaRa MutanBEST Kit, Takara Biotechnology, Dalian, China) with the corresponding primer pairs was used to generate sgRNA cassettes with new 20-bp complementary regions. Oligonucleotides used to generate sgRNA cassettes and the resultant sgRNA expression vectors are listed in Table 1.

### Construction of sgRNA-expressing plasmids to repress multiple genes

To repress multiple genes, different sgRNA sequences including the *Trc* promoter, 20-bp complementary region, 42-bp dCas9-binding hairpin and 40-bp transcription terminator were cloned into the same plasmid. The primer pair Pf_sgRNA(*Bam*HI)/Pr_sgRNA(*Eco*RI) was used to amplify anti-*adhE*, anti-*eno*, anti-*mdh*, anti-*fabB*, anti-*fumC* and anti-*sucC* sgRNA sequences into the *Bam*HI/*Eco*RI sites of pCOLA-fabF(high). This resulted in the plasmids pCOLA-fabF(high)/adhE(low), pCOLA-fabF(high)/eno(low), pCOLA-fabF(high)/mdh(low), pCOLA-fabF(high)/fabB(low), pCOLA-fabF(high)/fumC(medium) and pCOLA-fabF(high)/sucC(medium). The primer pair Pf_sgRNA(*Eco*RI)/Pr_sgRNA(*Hin*dIII) was used to amplify anti-*adhE*, anti-*eno*, anti-*fabB* and anti-*sucC* sgRNA sequences into the *Eco*RI/*Hin*dIII sites of pCOLA-fabF(high)/fumC(medium). This resulted in the plasmids pCOLA-fabF(high)/fumC(medium)/adhE(low), pCOLA-fabF(high)/fumC(medium)/eno(low), pCOLA-fabF(high)/fumC(medium)/fabB(low) and pCOLA-fabF(high)/fumC(medium)/sucC(medium). The primer pair Pf_sgRNA(*Hin*dIII)/Pr_sgRNA(*Nde*I) was used to amplify anti-*adhE*, anti-*eno* and anti-*sucC* sgRNA sequences into the *Hin*dIII/*Nde*I sites of pCOLA-fabF(high)/fumC(medium)/fabB(low). This resulted in the plasmids pCOLA-fabF(high)/fumC(medium)/fabB(low)/adhE(low), pCOLA-fabF(high)/fumC(medium)/fabB(low)/eno(low) and pCOLA-fabF(high)/fumC(medium)/fabB(low)/sucC(medium). The primer pair Pf_sgRNA(*Nde*I)/Pr_sgRNA(*Bgl*II) was used to amplify anti-*adhE* and anti-*eno* sgRNA sequences into the *Nde*I/*Bgl*II sites of pCOLA-fabF(high)/fumC(medium)/fabB(low)/sucC(medium). This resulted in the plasmids pCOLA-fabF(high)/fumC(medium)/fabB(low)/sucC(medium)/adhE(low) and pCOLA-fabF(high)/fumC(medium)/fabB(low)/sucC(medium)/eno(low). The primer pair Pf_sgRNA(*Bgl*II)/Pr_sgRNA(*Kpn*I) was used to amplify anti-*eno* sgRNA sequences into the *Bgl*II/*Kpn*I sites of pCOLA-fabF(high)/fumC(medium)/fabB(low)/sucC(medium)/adhE(low). This resulted in the plasmid pCOLA-fabF(high)/fumC(medium)/fabB(low)/sucC(medium)/adhE(low)/eno(low).

### Culture conditions

To investigate the silencing effects of various sgRNA species, cells were cultured in 25 mL of MOPS medium at 37 °C with 220 rpm orbital shaking at a starting OD_600_ of 0.1. After the OD_600_ reached 1.65, an additional 25 mL of fresh MOPS medium was added. sgRNA expression was induced with 0.5 mM IPTG. Cultures were subsequently incubated at 30 °C. 1 mL of cell culture was harvested at the mid-log phase of growth to quantify the intracellular concentrations of malonyl-CoA and acetyl-CoA. Final OD_600_ values of the cultures were measured after a total fermentation time of 48 h.

For (2S)-naringenin production, cells were first cultured in 25 mL of MOPS medium at 37 °C with 220 rpm orbital shaking at a starting OD_600_ of 0.1. After an OD_600_ of 1.65 had been reached, an additional 25 mL of fresh MOPS medium was added. Cultures were subsequently incubated at 30 °C for (2S)-naringenin production. IPTG and L-tyrosine were provided at final concentrations of 1 mM and 3 mM, respectively. After a total fermentation time of 48 h, 1 mL of cell culture was collected to measure the concentrations of (2S)-naringenin and *p*-coumaric acid^32^. Final OD_600_ values of the cultures were also measured after a total fermentation time of 48 h.

### Analytical methods

For each experiment, triplicate cultures were measured, and their deviation is represented by the error bar. To analyze (2S)-naringenin and *p*-coumaric acid production, *E. coli* cells were separated through centrifugation (5000 *g*, 15 min, 4 °C). 1 mL of supernatant was extracted with an equal volume of ethyl acetate. After vortexing and centrifugation (5000 *g*, 15 min, 4 °C), the top organic layer was separated and evaporated to dryness, and the remaining residue was resolubilized with 1 mL of methanol. Samples were analyzed by high-performance liquid chromatography (HPLC), using an Agilent 1100 series instrument and a reverse-phase Gemini NX-C18 column (5 × 110 mm) maintained at 25 °C. (2S)-Naringenin and *p*-coumaric acid were separated by elution with an acetonitrile/water gradient at a flow rate of 1.0 mL/min under the following conditions: 10 to 40% acetonitrile (vol/vol) for 10 min, 40% acetonitrile (vol/vol) for 5 min, 40 to 10% acetonitrile (vol/vol) for 2 min^24^.

To quantify the malonyl-CoA concentration, an aliquot of 1 mL of cell culture was removed and chilled immediately on ice and centrifuged at 5000 *g* and 4 °C for 10 min. The cell pellet was resuspended in 1 mL of 6% perchloric acid to facilitate cell lysis. The lysed cell suspension was then neutralized with 0.3 mL of 3 M potassium carbonate while vortexing to neutralize the acid. The solution was centrifuged to pellet the cell debris. The supernatant was collected and stored chilled until the malonyl-CoA content was analyzed. To determine the dry cell weight, 2 mL of the same culture were filtered through a 0.45-μm cellulose membrane (Sangon Biotech, Shanghai, China), followed by washing with distilled water and drying in a conventional oven. Dry cell weight was represented by the weight difference between empty membranes and those with cell residues^5^.

Malonyl-CoA and acetyl-CoA from the cell cultures were identified by the area of major mass spectra signals ([M–H]^−^). A liquid chromatography-mass spectrophotometer (Shimadzu, Kyoto, Japan) equipped with an electrospray ionization (ESI) source and a reverse-phase Gemini NX-C18 column (5 × 110 mm) was used. A Shim-Pack VP-ODS 150L × 2.0 HPLC column (Shimadzu, Kyoto, Japan) was used to perform HPLC separation. Samples of malonyl-CoA and acetyl-CoA were eluted at a flow rate of 0.3 mL/min with a gradient of 15 mM ammonium formate (A) and 10% 10 mM ammonium acetate in methanol (v/v) (B) as follows: at 0 min, 2% B; 10 min, 60% B; 20 min, 76% B; 25 min, 2% B. The MS/MS system was operated in negative ion mode using optimized conditions: detector voltage, 1.60 Kv; nebulizing gas (N_2_) flow, 1.5 L/min; drying gas (N_2_) flow, 200 kPa; ion accumulation time, 30 ms; scan range m/z, 100–1000 for MS^1^, 100–500 for MS^2^.

## Additional Information

**How to cite this article**: Wu, J. *et al.* Enhancing flavonoid production by systematically tuning the central metabolic pathways based on a CRISPR interference system in *Escherichia coli*. *Sci. Rep.* **5**, 13477; doi: 10.1038/srep13477 (2015).

## Supplementary Material

Supplementary Information

## Figures and Tables

**Table 1:**
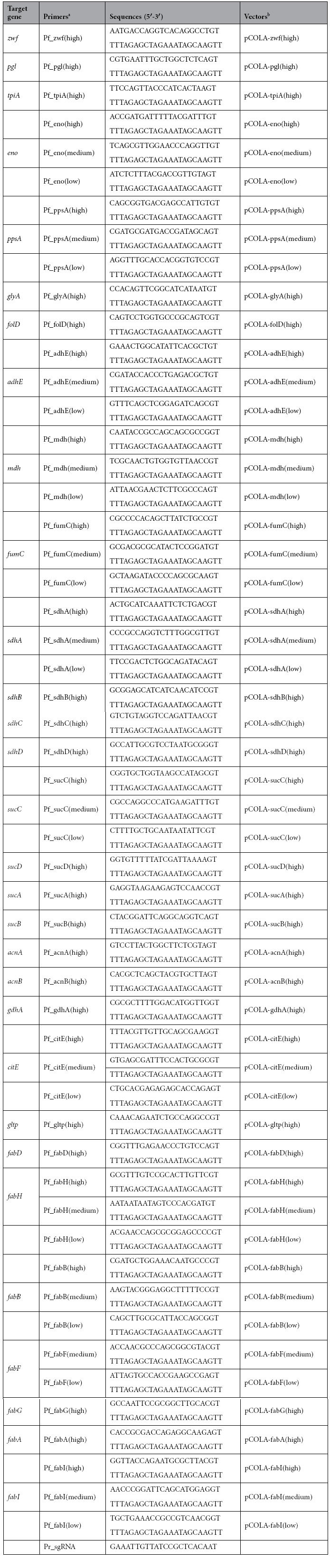
Primers and vectors used for single gene perturbations.

^a^Each primer pair shares the same reverse primer Pr_sgRNA (at the end of the Table).

^b^‘High’, ‘Medium’ and ‘Low’ mean constructed vectors with high, medium and low silencing efficacy toward target genes, respectively.

**Table 2:** Primers used for multiple gene perturbations.

**Oligonucleotides**	Sequences (5′-3′)*
Pf_sgRNA(*Bam*HI)	CGC**GGATCC**TGTACACTGCAGGTCGTAAATCAC
Pr_sgRNA(*Eco*RI)	CCG**GAATTC**AAAAAAGCACCGACTCGGTG
Pf_sgRNA(*Eco*RI)	CCG**GAATTC**TGTACACTGCAGGTCGTAAATCAC
Pr_sgRNA(*Hin*dIII)	CCC**AAGCTT**AAAAAAGCACCGACTCGGTG
Pf_sgRNA(*Hin*dIII)	CCC**AAGCTT**TGTACACTGCAGGTCGTAAATCAC
Pr_sgRNA(*Nde*I)	GGAATTC**CATATG**AAAAAAGCACCGACTCGGTG
Pf_sgRNA(*Nde*I)	GGAATTC**CATATG**TGTACACTGCAGGTCGTAAATCAC
Pr_sgRNA(*Bgl*II)	GA**AGATCT**AAAAAAGCACCGACTCGGTG
Pf_sgRNA(*Bgl*II)	GA**AGATCT**TGTACACTGCAGGTCGTAAATCAC
Pr_sgRNA(*Kpn*I)	GG**GGTACC**AAAAAAGCACCGACTCGGTG

^*^Bold and underlined letters are restriction enzyme cut sites.

**Table 3:** Plasmids used for multiple gene perturbations.

**Plasmids**	**Description**	**Sources**
pCDFDuet-1	Double *T7* promoters, CDF ori, Sm^R^	Novagen
pETDuet-1	Double *T7* promoters, pBR322 ori, Amp^R^	Novagen
pACYCDuet-1	Double *T7* promoters, P15A ori, Cm^R^	Novagen
pCOLADuet-1	Double *T7* promoters, ColA ori, Kn^R^	Novagen
pCDF-Trc-TAL-Trc-4CL	pCDFDuet-1 carrying TAL and 4CL under *Trc* promoter	24
pET-CHS-CHI	pETDuet-1 carrying CHS and CHI	24
pACYC-dCas9	pACYCDuet-1 carrying dCas9	This study
pCOLA-fabF(high)/adhE(low)	Vectors with high silencing efficacy toward *fabF* and low silecncing efficacy toward *adhE*	This study
pCOLA-fabF(high)/eno(low)	Vectors with high silencing efficacy toward *fabF* and low silecncing efficacy toward *eno*	This study
pCOLA-fabF(high)/mdh(low)	Vectors with high silencing efficacy toward *fabF* and low silecncing efficacy toward *mdh*	This study
pCOLA-fabF(high)/fabB(low)	Vectors with high silencing efficacy toward *fabF* and low silecncing efficacy toward *fabB*	This study
pCOLA-fabF(high)/fumC(medium)	Vectors with high silencing efficacy toward *fabF* and medium silecncing efficacy toward *fumC*	This study
pCOLA-fabF(high)/sucC(medium)	Vectors with high silencing efficacy toward *fabF* and medium silecncing efficacy toward *sucC*	This study
pCOLA-fabF(high)/fumC(medium)/adhE(low)	Vectors with high, medium and low silencing efficacy toward *fabF*, *fumC* and *adhE*, respectively	This study
pCOLA-fabF(high)/fumC(medium)/eno(low)	Vectors with high, medium and low silencing efficacy toward *fabF*, *fumC* and *eno*, respectively	This study
pCOLA-fabF(high)/fumC(medium)/fabB(low)	Vectors with high, medium and low silencing efficacy toward *fabF*, *fumC* and *fabB*, respectively	This study
pCOLA-fabF(high)/fumC(medium)/sucC(medium)	Vectors with high, medium and medium silencing efficacy toward *fabF*, *fumC* and *sucC*, respectively	This study
pCOLA-fabF(high)/fumC(medium)/fabB(low)/adhE(low)	Vectors with high, medium, low and low silencing efficacy toward *fabF*, *fumC*, *fabB* and *adhE*, respectively	This study
pCOLA-fabF(high)/fumC(medium)/fabB(low)/eno(low)	Vectors with high, medium, low and low silencing efficacy toward *fabF*, *fumC*, *fabB* and *eno*, respectively	This study
pCOLA-fabF(high)/fumC(medium)/fabB(low)/sucC(medium)	Vectors with high, medium, low and medium silencing efficacy toward *fabF*, *fumC*, *fabB* and *sucC*, respectively	This study
pCOLA-fabF(high)/fumC(medium)/fabB(low)/sucC(medium)/adhE(low)	Vectors with high, medium, low, medium and low silencing efficacy toward *fabF*, *fumC*, *fabB*, *sucC* and *adhE*, respectively	This study
pCOLA-fabF(high)/fumC(medium)/fabB(low)/sucC(medium)/eno(low)	Vectors with high, medium, low, medium and low silencing efficacy toward *fabF*, *fumC*, *fabB*, *sucC* and *eno*, respectively	This study
pCOLA-fabF(high)/fumC(medium)/fabB(low)/sucC(medium)/adhE(low)/eno(low)	Vectors with high, medium, low, medium, low, low and low silencing efficacy toward *fabF*, *fumC*, *fabB*, *sucC*, *adhE* and *eno*, respectively	This study
