# Supplementary material for: Enhancing flavonoid production by systematically tuning the central metabolic pathways based on a CRISPR interference system in Escherichia coli
Source: Sci Rep. 2015 Sep 1;5:13477. doi: 10.1038/srep13477 (PMC4555050; doi:10.1038/srep13477)
Supplement: Supplementary Information [file srep13477-s1.pdf]

Enhancing flavonoid production by systematically tuning the central metabolic pathways based on a CRISPR interference system in *Escherichia coli*

Junjun Wu[1,2], Guocheng Du[1,2], Jian Chen[1,2], Jingwen Zhou[1,2*]

[1] Key Laboratory of Industrial Biotechnology, Ministry of Education, School of Biotechnology, Jiangnan University, 1800 Lihu Road, Wuxi, Jiangsu 214122, China;

[2] Synergetic Innovation Center of Food Safety and Nutrition, 1800 Lihu Road, Wuxi, Jiangsu 214122, China.

[*] Corresponding authors.

Jingwen Zhou

Mailing address: School of Biotechnology, Jiangnan University, 1800 Lihu Road, Wuxi, Jiangsu 214122, China

Phone: +86-510-85329031, Fax: +86-510-85918309

E-mail: zhoujw1982@jiangnan.edu.cn

DNA sequences of optimized genes

Gene sequence of dCas9 protein

ATGGACAAAAAATACAGCATCGGTCTGGCAATCGGTACGAACTCTGTTGG
CTGGGCGGTTATCACTGATGAATACAAAGTCCCGTCCAAAAAATTCAAAG
TGCTGGGCAACACCGATCGTCACTCCATCAAGAAAAACCTGATTGGCGCG
CTGCTGTTCGACTCCGGTGAAACTGCTGAAGCGACCCGCCTGAAACGTAC
TGCACGCCGTCGTTACACCCGCCGTAAGAACCGCATTTGCTACCTGCAGG
AAATTTTCAGCAACGAAATGGCGAAAGTAGACGACAGCTTTTTCCACCGT
CTGGAGGAGAGCTTTCTGGTTGAAGAAGACAAAAAACACGAGCGCCACC
CTATTTTCGGCAACATTGTGGACGAAGTGGCATACCACGAAAAATACCCG
ACCATCTACCACCTGCGTAAAAAACTGGTTGATAGCACCGACAAAGCAGA
CCTGCGTCTGATCTACCTGGCGCTGGCCCACATGATCAAATTCCGTGGCCA
CTTCCTGATCGAAGGCGATCTGAACCCAGACAACTCTGACGTGGACAAAC
TGTTTATCCAGCTGGTGCAGACCTATAACCAGCTGTTTGAAGAGAACCCG
ATCAACGCTTCTGGCGTTGATGCTAAAGCCATCCTGTCTGCTCGTCTGTCT
AAATCCCGTCGTCTGGAGAATCTGATTGCTCAGCTGCCGGGCGAAAAGAA
AAACGGTCTGTTTGGCAACCTGATCGCGCTGTCCCTGGGCCTGACTCCGAA
CTTCAAATCTAACTTCGACCTGGCTGAAGATGCAAAACTGCAACTGTCCA
AAGACACTTATGACGATGATCTGGATAACCTGCTGGCGCAGATCGGTGAT
CAGTATGCAGACCTGTTCCTGGCTGCCAAAAACCTGTCTGATGCTATCCTG
CTGAGCGACATCCTGCGCGTTAACACTGAAATCACCAAAGCTCCGCTGTC
TGCGTCTATGATCAAACGCTATGACGAACATCACCAGGACCTGACCCTGC

TGAAAGCGCTGGTACGTCAGCAACTGCCGGAAAAATACAAAGAAATCTTC
TTCGACCAGAGCAAAAACGGCTACGCGGGTTATATCGATGGCGGTGCTAG
CCAGGAAGAGTTCTACAAATTCATCAAGCCGATCCTGGAAAAAATGGATG
GTACTGAAGAGCTGCTGGTTAAACTGAACCGTGAAGACCTGCTGCGTAAA
CAGCGTACTTTCGACAATGGCTCTATTCCGCACCAGATTCATCTGGGTGAA
CTGCATGCAATTCTGCGCCGTCAGGAGGATTTCTATCCGTTTCTGAAAGAT
AATCGCGAAAAAATCGAAAAAATTCTGACTTTCCGTATTCCGTACTACGT
GGGTCCGCTGGCGCGTGGTAATTCCCGCTTTGCATGGATGACCCGTAAATC
TGAAGAAACCATTACCCCTTGGAACTTCGAAGAAGTCGTTGATAAAGGTG
CTTCTGCACAGTCTTTCATCGAACGCATGACGAATTTCGACAAAAATCTGC
CGAACGAGAAAGTACTGCCGAAGCATTCCCTGCTGTACGAATACTTCACC
GTCTATAACGAACTGACGAAAGTTAAATACGTAACCGAAGGTATGCGCAA
ACCGGCCTTTCTGTCCGGTGAGCAGAAGAAAGCCATCGTGGACCTGCTGT
TTAAAACCAACCGCAAAGTAACCGTAAAACAACTGAAAGAAGACTACTTT
AAGAAAATCGAATGTTTTGATAGCGTTGAAATTTCCGGTGTTGAAGATCG
TTTCAACGCCTCTCTGGGCACGTATCACGATCTGCTGAAGATTATCAAAGA
TAAAGACTTTCTGGACAACGAAGAGAACGAAGACATTCTGGAGGACATCG
TTCTGACGCTGACGCTGTTCGAAGATCGTGAGATGATCGAAGAACGTCTG
AAAACTTACGCTCATCTGTTCGACGATAAAGTCATGAAACAGCTGAAACG
TCGTCGTTATACGGGTTGGGGTCGCCTGTCTCGCAAGCTGATTAACGGCAT
CCGCGATAAACAATCTGGCAAAACCATCCTGGATTTCCTGAAAAGCGATG
GCTTCGCAAACCGTAACTTCATGCAGCTGATTCATGACGATTCTCTGACCT

64 TCAAAGAAGATATTCAAAAAGCGCAAGTTTCCGGTCAAGGCGACTCCCTG

65 CACGAACACATCGCCAACCTGGCGGGTTCTCCAGCTATCAAGAAAGGCAT

66 CCTGCAAACTGTAAAAGTTGTTGACGAACTGGTTAAAGTTATGGGCCGTC

67 ACAAACCGGAAAACATCGTGATTGAAATGGCACGTGAGAACCAGACCAC

68 CCAGAAAGGTCAGAAAAACTCTCGTGAACGTATGAAACGCATTGAAGAA

69 GGTATCAAAGAGCTGGGCTCCCAAATCCTGAAAGAACACCCGGTCGAAAA

70 CACTCAGCTGCAGAACGAAAAACTGTACCTGTATTACCTGCAGAATGGTC

71 GTGACATGTATGTTGACCAGGAACTGGACATCAACCGTCTGTCCGACTAC

72 GACGTCGACGCAATCGTGCCGCAGTCTTTCCTGAAGGATGACTCTATCGA

73 CAACAAAGTTCTGACTCGCAGCGATAAAAACCGCGGCAAAAGCGATAAC

74 GTTCCGTCCGAAGAAGTTGTGAAAAAGATGAAAAACTATTGGCGTCAGCT

75 GCTGAATGCCAAACTGATTACCCAGCGCAAATTTGATAACCTGACCAAAG

76 CGGAACGTGGTGGTCTGTCCGAACTGGATAAAGCAGGTTTCATTAAACGT

77 CAGCTGGTAGAGACGCGTCAGATCACTAAGCACGTGGCTCAGATCCTGGA

78 CTCTCGTATGAACACCAAATATGACGAAAATGATAAGCTGATTCGTGAAG

79 TAAAGGTGATCACTCTGAAAAGCAAGCTGGTCTCCGATTTCCGCAAAGAT

80 TTCCAGTTTTACAAAGTGCGTGAGATCAACAACTACCACCATGCGCACGA

81 TGCGTATCTGAACGCTGTCGTTGGCACCGCACTGATCAAGAAATACCCAA

82 AGCTGGAAAGCGAGTTCGTGTATGGTGATTATAAAGTGTATGACGTACGT

83 AAAATGATCGCGAAGTCTGAACAGGAAATCGGCAAAGCTACCGCCAAGT

84 ACTTCTTTTACTCCAACATTATGAACTTCTTCAAAACCGAAATCACCCTGG

85 CTAATGGCGAGATCCGCAAGCGCCCTCTGATTGAAACTAACGGTGAAACC

GGCGAAATCGTATGGGATAAAGGTCGCGATTTCGCGACGGTACGTAAAGT
CCTGTCCATGCCGCAGGTTAACATTGTTAAAAAGACCGAAGTTCAGACCG
GTGGTTTTTCCAAAGAATCCATCCTGCCGAAACGTAACAGCGACAAACTG
ATCGCCCGCAAAAAGGACTGGGATCCAAAGAAATACGGTGGTTTCGACTC
CCCGACCGTTGCTTATTCTGTTCTGGTTGTGGCCAAAGTGGAGAAGGGTAA
AAGCAAGAAACTGAAATCTGTTAAAGAACTGCTGGGCATCACCATCATGG
AGCGTAGCTCCTTTGAGAAAAACCCTATTGACTTCCTGGAAGCAAAAGGC
TACAAAGAAGTAAAGAAGGACCTGATCATTAAACTGCCGAAATATAGCCT
GTTCGAACTGGAAAACGGTCGTAAACGTATGCTGGCATCTGCGGGCGAAC
TGCAGAAAGGCAACGAACTGGCTCTGCCTTCTAAATACGTGAACTTCCTG
TACCTGGCGTCTCATTACGAAAAGCTGAAAGGCAGCCCAGAGGATAACGA
GCAAAAGCAGCTGTTCGTGGAACAGCACAAACACTACCTGGATGAGATCA
TTGAACAGATCTCCGAGTTCTCTAAACGTGTAATCCTGGCGGACGCGAAT
CTGGACAAAGTACTGTCCGCATACAATAAACACCGTGATAAACCGATCCG
TGAACAGGCTGAGAACATCATCCATCTGTTCACTCTGACTAACCTGGGCG
CGCCGGCTGCATTTAAGTACTTCGACACCACCATCGATCGTAAGCGTTACA
CTAGCACCAAAGAAGTGCTGGACGCGACCCTGATTCACCAGAGCATCACT
GGTCTGTACGAAACCCGCATTGATCTGTCTCAGCTGGGTGGTGACTAA

Gene sequence of sgRNA chimera

TGTACACTGCAGGTCGTAAATCACTGCATAATTCGTGTCGCTCAAGGCGC
ACTCCCGTTCTGGATAATGTTTTTTGCGCCGACATCATAACGGTTCTGGCA

AATATTCTGAAATGAGCTGTTGACAATTAATCATCCGGCTCGTATAATGTGTGGAATTGTGAGCGGATAACAATTTCAGGAGACAACATGCCCAGTCGTTTTAGAGCTAGAAATAGCAAGTTAAAATAAGGCTAGTCCGTTATCAACTTGAAAAAGTGGCACCGAGTCGGTGCTTTTTT

Supplementary Table

Table S1 The specific sequences of sgRNA targeted in the initial, intermediate, or terminal gene coding region in target genes

Target gene	Target region*	Sequences (5'-3')
zwf	Initial	AATGACCAGGTCACAGGCCT
pgl	Initial	CGTGAATTTGCTGGCTCTCA
tpiA	Initial	TTCCAGTTACCCATCACTAA
	Initial	ACCGATGATTTTTACGATTT
eno	Intermediate	TCAGCGTTGGAACCCAGGTT
	Terminal	ATCTCTTTACGACCGTTGTA
	Initial	CAGCGGTGACGAGCCATTGT
ppsA	Intermediate	CGATGCGATGACCGATAGCA
	Terminal	AGGTTTGCACCACGGTGTCC
glyA	Initial	CCACAGTTCGGCATCATAAT
folD	Initial	CAGTCCTGGTGCCCGCAGTC
adhE	Initial	GAAACTGGCATATTCACGCT
	Intermediate	CGATACCACCCTGAGACGCT

	Terminal	GTTTCAGCTCGGAGATCAGC
	Initial	CAATACCGCCAGCAGCGCCG
mdh	Intermediate	TCGCAACTGTGGTGTTAACC
	Terminal	ATTAACGAACTCTTCGCCCA
	Initial	CGCCCCACAGCTTATCTGCC
fumC	Intermediate	GCGACGCGCATACTCCGGAT
	Terminal	GCTAAGATACCCCAGCGCAA
	Initial	ACTGCATCAAATTCTCTGAC
sdhA	Intermediate	CCCGCCAGGTCTTTGGCGTT
	Terminal	TTCCGACTCTGGCAGATACA
sdhB	Initial	GCGGAGCATCATCAACATCC
sdhC	Initial	GTCTGTAGGTCCAGATTAAC
sdhD	Initial	GCCATTGCGTCCTAATGCGG
	Initial	CGGTGCTGGTAAGCCATAGC
sucC	Intermediate	CGCCAGGCCCATGAAGATTT
	Terminal	CTTTTGCTGCAATAATATTC
sucD	Initial	GGTGTTTTTATCGATTAAAA
sucA	Initial	GAGGTAAGAAGAGTCCAACC
sucB	Initial	CTACGGATTCAGGCAGGTCA
acnA	Initial	GTCCTTACTGGCTTCTCGTA
acnB	Initial	CACGCTCAGCTACGTGCTTA
gdhA	Initial	CGCGCTTTTGGACATGGTTG

	Initial	TTTACGTTGTTGCAGCGAAG
citE	Intermediate	GTGAGCGATTTCCACTGCGC
	Terminal	CTGCACGAGAGAGCACCAGA
gltp	Initial	CAAACAGAATCTGCCAGGCC
fabD	Initial	CGGTTTGAGAACCCTGTCCA
	Initial	GCGTTTGTCCGCACTTGTTC
fabH	Intermediate	AATAATAATAGTCCCACGAT
	Terminal	ACGAACCAGCGCGGAGCCCC
	Initial	CGATGCTGGAAACAATGCCC
fabB	Intermediate	AAGTACGGGAGGCTTTTTCC
	Terminal	CAGCTTGCGCATTACCAGCG
fabF	Intermediate	ACCAACGCCCAGCGGCGTAC
	Terminal	ATTAGTGCCACCGAAGCCGA
fabG	Initial	GCCAATTCCGCGGCTTGCAC
fabA	Initial	CACCGCGACCAGAGGCAAGA
	Initial	GGTTACCAGAATGCGCTTAC
fabI	Intermediate	AACCCGGATTCAGCATGGAG
	Terminal	TGCTGAAACCGCCGTCAACG
